# The Diagnosis and Treatment of Local Complications of Acute Necrotizing Pancreatitis in China: A National Survey

**DOI:** 10.1155/2021/6611149

**Published:** 2021-07-10

**Authors:** Xiaowu Dong, Wenjian Mao, Lu Ke, Lin Gao, Jing Zhou, Bo Ye, Gang Li, Anthony Phillips, Zhihui Tong, John Windsor, Weiqin Li, Chinese Acute Pancreatitis Clinical Trials Group (CAPCTG)

**Affiliations:** ^1^Center of Severe Acute Pancreatitis (CSAP), Department of Critical Care Medicine, Jinling Clinical Medical College of Nanjing Medical University, No. 305 Zhongshan East Road, Nanjing, China; ^2^Center of Severe Acute Pancreatitis (CSAP), Department of Critical Care Medicine, Jinling Hospital, Medical School of Nanjing University, No. 305 Zhongshan East Road, Nanjing, China; ^3^Surgical and Translational Research Centre, Faculty of Medical and Health Sciences, University of Auckland, Auckland, New Zealand; ^4^Applied Surgery and Metabolism Laboratory, School of Biological Sciences, University of Auckland, Auckland, New Zealand; ^5^HBP/Upper GI Unit, Department of General Surgery, Auckland City Hospital, Auckland, New Zealand; ^6^National Institute of Healthcare Data Science at Nanjing University, Nanjing, China

## Abstract

**Background:**

The optimal management strategy in acute necrotizing pancreatitis (ANP) is debated, and compliance with current guidelines in China is not known. In this study, we performed a national survey on this topic in China.

**Methods:**

An online questionnaire about the diagnosis and treatment of local complications of ANP was distributed through a national collaborative network. The local and systemic complications were defined according to the Revised Atlanta Classification.

**Results:**

There were 321 survey respondents from the 394 who opened the link (response rate 81%) from 208 hospitals located in 30/34 provinces across China. There was a lack of consensus in terms of early diagnosis of infected pancreatic necrosis (IPN) as the respondents chose to depend on clinical symptoms (70/321, 22%), organ failure (82/321, 26%), imaging changes (84/321, 26%), and fine needle aspiration (51/321, 16%), respectively. A “step-up” approach has been widely adopted in patients with IPN (294/321, 92%). The decision for initial intervention (without confirmed/suspected infection) was based on clinical condition, CT imaging, or laboratory indicators for most respondents (229/321, 71%).

**Conclusion:**

While the “step-up” approach has been widely adopted, there is still significant variation in regard to the diagnosis of infection, the best timing for drainage, and the indications for early intervention.

## 1. Introduction

Acute pancreatitis (AP) is one of the most common gastrointestinal disorders and a major cause of acute hospital admission in China [1]. Pancreatic necrosis occurs in 5-10% of all AP episodes [2]. The most important determinant of severity in patients with AP is persistent organ failure (POF) [3] which can account for more than half of the deaths from AP [4, 5]. The local complications of AP are responsible for considerable morbidity and have been redefined in the Revised Atlanta Classification (RAC) [2, 6], and there has been a notable evolution in their treatment over the last a few years [7].

The early treatment of severe AP is mostly supportive with the primary aim of correcting intravascular volume depletion and organ support (e.g., vasopressors, mechanical ventilation, and renal replacement). The treatment of acute local complications of AP, like acute pancreatic fluid collections (APFC) and acute necrotic collections (ANC), is rarely indicated in the acute setting [8, 9]. The local complications can get infected in approximately a third of patients with ANP and therefore become infected pancreatic necrosis (IPN). IPN is a challenging and potentially fatal complication, with a mortality of 15-30% [10–12]. Several criteria for the diagnosis of IPN have been reported, but the optimal diagnostic strategy remains uncertain [8, 13–16]. The approaches include the development of clinical markers of infection, laboratory markers (including procalcitonin), the detection of gas within the collection on contrast-enhanced computed tomography (CECT), and positive fine needle aspiration (FNA) culture results.

The current recommended treatment of confirmed IPN is antibiotics and drainage (percutaneous or endoscopic). This has replaced the “surgery-first” approach with necrosectomy and drainage [17]. The “step-up” approach of drainage first (followed by endoscopic or percutaneous debridement of necrosis) has gradually become the standard of treatment in recent years, although uncertainty remains around the optimal timing, drainage, and debridement techniques [8, 11, 12, 18]. This means that there is a significant variation in the management strategies of AP between different centers or even within centers. Further, it is not known to what extent the current evidence for the “step-up” approach has been implemented in China. Therefore, the aim of this study was to determine the current diagnostic and treatment strategies for local complications of ANP in China.

## 2. Materials and Methods

An online 11-question survey (Table 1) was distributed to Chinese doctors using a commercial platform tool through the Acute Pancreatitis Network (APnet) which is a web-based platform designed to promote education and research for health professionals involved in the care of patients with AP [19]. The subscribers are based in four different medical specialties, intensive care medicine, emergency medicine, gastroenterology, and general surgery. Both the local complications like ANC, APFC, walled-off necrosis (WON), and IPN and systemic complications like organ failure and persistent organ failure were defined according to the RAC terminology [2].

The survey was conducted over a 4-week period (July 9^th^, 2018~Aug 5^th^, 2018), with the invitation to participate issued via email, a notice on the APnet, and social media (WeChat, Tencent, Shenzhen China) with weekly reminders. The physicians conducting the survey were excluded from this survey. This was a “closed” survey, and participants were required to log in first to prevent duplicate entries. It was also a voluntary survey without any incentives offered. There were 11 questions in the survey including 2 adaptive question. Question 1.1 was displayed based on responses to question 1, and question 1.1.1 based on question 1.1. Completeness would be checked by the website before the questionnaire was submitted. Participants were able to review and change their answers through a Back button before submitting.

Data were collected anonymously and analyzed using IBM SPSS 24.0 software. Only completed questionnaires would be analyzed. The data were categorical variables and described as counts and percentages. All methods were carried out under the relevant guideline [20]. Approval was obtained from the institutional review board of the Jinling Hospital, and informed consent was incorporated into the questionnaire and signed prior to answering the questions.

## 3. Results

### 3.1. Characteristics of Respondents and Institutions

**Figure 1:**
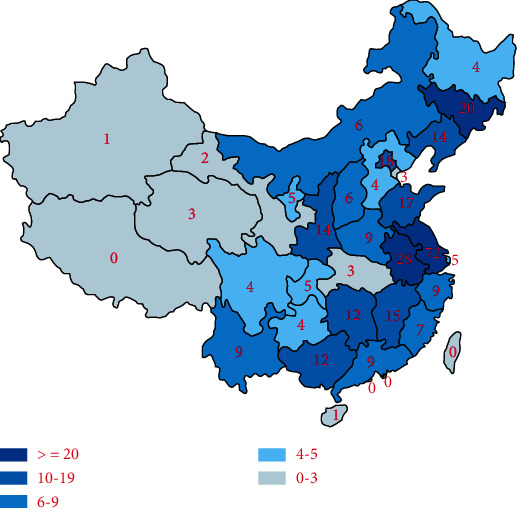
Geographic distribution of the respondents.

**Figure 2:**
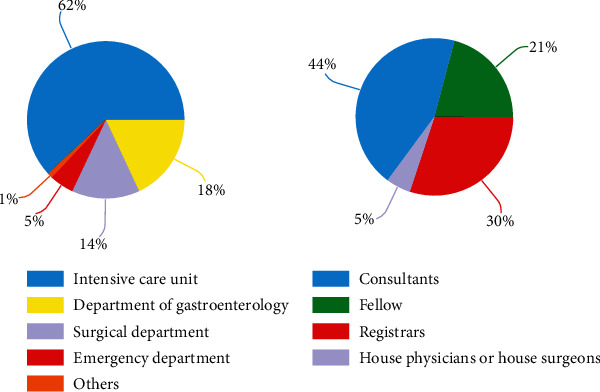
Baseline characteristics of respondents.

There were 321 survey responders who participated and completed the survey from the 394 who opened the link (response rate 81%, completion rate 100%) from 208 hospitals distributed in 30/34 provinces across China (Figure 1). A vast majority of the participating institutes were tertiary ones or above (88%, 183/208), including 14 quaternary hospitals, and the rest 25 (12%) were secondary hospitals. The respondents were from intensive care medicine (62%, 197/321), gastroenterology (18%, 59/321), general surgery (14%, 44/321), emergency medicine (5%, 17/321), and other departments (1%, 4/321) (Figure 2). There was a wide distribution of seniority among the respondent: consultants (44%, 139/321), fellows (21%, 67/321), registrars (30%, 98/321), and house officers (5%, 15/321) (Figure 2).

### 3.2. Treatment Strategy for Sterile ANC

The decision to drain patients without signs of infection was based on clinical condition, CT imaging, or laboratory indicators for 71% (*N* = 229) respondents, while 18% (*N* = 57) indicated they would never consider drainage, and 11% (*N* = 35) said they would offer immediate drainage whenever possible (Table 1). Of the 229 respondents who would drain “based on clinical condition, CT imaging, or laboratory indicators,” most (*N* = 126, 55%) were mainly concerned about organ failure (OF), while for 17% (*N* = 39), it was persistent abdominal pain or abdominal distension, 15% (*N* = 34) CT evidence of increasing extent of pancreatic/peripancreatic necrosis, and 8% (*N* = 18) laboratory indicators. Among the 126 doctors who would offer drainage because of OF, 45% (*N* = 57) would do so for POF, 42% (*N* = 53) for deteriorating original OF, 33% (*N* = 41) for new-onset OF, 17% (*N* = 21) for OF at least 1 week, and 7% (*N* = 9) for OF of 2-week duration or more.

Interventions for patients without signs of infection included percutaneous catheter drainage (PCD) in 47% (*n* = 153), percutaneous or endoscopic drainage depending on the location of the ANC in 42% (*N* = 135), endoscopic drainage in 4% (*N* = 12), surgical drainage in 4% (*N* = 12), and other drainage technique in 3% (*N* = 9). 57% of the respondents (*N* = 33) from gastroenterology would choose percutaneous or endoscopic drainage depending on the location, while respondents from other departments preferred PCD (Supplement Table 1).

### 3.3. Treatment Strategy for Demarcated Pancreatic Necrosis

For the WON with symptoms but no suspicion of infection, 82% (*N* = 262) of the respondents would take a step-up approach starting with drainage (percutaneous or endoscopic), while 14% (*N* = 45) would opt for open surgical debridement/drainage and 4% (*N* = 14) would choose other treatments, including observation without drainage (Table 1).

### 3.4. Diagnosis of Infection of Pancreatic Necrosis

What was considered most important in suspected infection of a local complication of AP was variable: 26% (*N* = 84) considered changes in CT or MR imaging, 26% (*N* = 82) stated new-onset OF or deteriorated existing OF, 22% (*N* = 70) of the respondents considered spiking of body temperatures to greater than 38.5°C, 16% (*N* = 51) required diagnosis by fine needle aspiration, 8% (*N* = 27) based it on laboratory indicators, and 2% (*N* = 7) others. Very few respondents (8%, *N* = 25) would perform fine needle aspiration routinely in response to suspicion of IPN, 40% (*N* = 130) would never do it, while 52% (*N* = 166) would do it selectively (Table 1).

### 3.5. Treatment Strategy for Infected Complications of AP

Patients with confirmed infection would be offered drainage if the collection was well demarcated or walled off for 35% (*N* = 112) of the respondents, whereas 25% (*N* = 79) would base the decision on the lack of satisfactory response to antibiotic treatment, 22% (*N* = 72) would undertake immediate drainage for confirmed infection and would not consider other factors, and 14% (*N* = 45) for occurrence or evolution of OF, and 4% (*N* = 13) for other reasons, including a positive blood culture (Table 1).

For patients with confirmed IPN but an immature (not well demarcated or encapsulated) collection, 50% (*N* = 162) of respondents would administer antibiotics and drain (percutaneous or endoscopic) with a view to performing a delayed necrosectomy after 4 weeks, while 25% (*N* = 79) would administer antibiotics and wait for demarcation, and 16% (*N* = 79) would administer antibiotics and only consider drainage if antibiotics failed. Immediate debridement (endoscopic or surgical) was favored by only 5% (*N* = 16) respondents (Table 1).

For patients with confirmed IPN and a mature (well demarcated, encapsulated, or walled off) collection, 78% (*N* = 250) of respondents would administer antibiotics and arrange immediate drainage (percutaneous or endoscopic), 8% (*N* = 25) would not consider drainage until antibiotics had proven futile, and 8% (*N* = 25) would administer antibiotics and arrange immediate minimally invasive debridement (Table 1).

The preferred drainage strategy for confirmed IPN was the step-up approach for 92% (*N* = 294) of the respondents. This would be with either percutaneous or endoscopic drainage based on the collection locations and available expertise. Only 5% (*N* = 15) would consider open surgical debridement and drainage (Table 1).

## 4. Discussion

This is the first national survey in China to document the approach to the diagnosis and treatment of the local complications of ANP. The results highlight significant variation in practice across China while also demonstrating the significant uptake of the “step-up” approach [21], which has been shown in other studies to be associated with a significantly reduced requirement for open surgical treatment and improved outcomes [22, 23].

The survey was distributed widely to doctors in China using the web-based platform of the Acute Pancreatitis Network, which encompasses 4 specialties (general surgery, intensive care medicine, emergency medicine, gastroenterology) and 3511 centers. Responses were obtained from hospitals located in 30 of the 34 provincial regions (except Tibet, Hongkong SAR, Taiwan, China, and Macau SAR). 394 subscribers logged onto the survey, of whom 321 responded (response rate 81%).

The indication and timing of intervention for sterile local complications of AP were a marked lack of consensus. The reasons for intervention were largely based on organ failure, and this included persistent, worsening, and new onset of organ failure. It was notable that the majority of respondents supported early intervention in this setting even in the absence of any recommendations for this in current guidelines [11, 22]. The International Pancreas Association and the American Pancreas Association jointly published guidelines recommending that when ANC has explicitly excluded infections, if organ failure persists for several weeks after onset, intervention therapy may be considered, but preferably not until ANC evolves to WON [11]. In the latest consensus published by the European Endoscopy Association, it was suggested that invasive intervention was indicated for patients with POF or “failure to thrive” over several weeks, but the concepts of “several weeks” and “failure to thrive” are still ambiguous [22]. More recently, a small study showed some benefit of early aggressive drainage of sterile ANC in reducing SIRS and POF and for the early diagnosis of IPN [23].

The optimal approach to the diagnosis of infected local complications of AP is important for deciding about intervention, but this remains controversial, and there was no consensus [24]. Only 26% (*N* = 84) of the respondents thought that gas within the local complication/collection was the most important diagnostic criteria. Collection with gas had high specificity but poor sensitivity. Previous study showed that gas were seen in only 42% of patients with infected necrosis [25]. Image-guided FNA-bacterial culture was previously widely used to confirm the suspicion of infection, but most experts rarely or never use it [24, 25] in part because of the 12–25% risk of false-negative result [26, 27] and the risk of introducing infection. Clinical and laboratory markers can be abnormal for other reasons, including a sterile systemic inflammatory response and extrapancreatic infections [28]. After the first 14 days, clinical signs of infected necrosis are much more reliable. It is possible to attain high levels of accuracy in the identification of infected necrosis based on clinical criteria [29]. Without an early and accurate approach to the definitive diagnosis of IPN, intervention is based on the probability of infection using clinical, radiological, and biochemical methods, highlighting a significant knowledge gap. The results of this survey indicate that 71% (229/321) respondents would drain patients without proven infection based on concerns about clinical condition, CT imaging, or laboratory indicators, and this would be based on organ failure (persistent, new-onset, and deteriorating) in 55%,126/229 respondents. This is despite the lack of evidence that draining a sterile ANC is of benefit and acceptable risk. And it is not known what proportion of these patients would prove to have infection and how many would have infection introduced by the procedure. One of the advantages of a more liberal approach to drainage in a patient that is deteriorating, whether it is on the basis of cytokine or infection mediated organ failure, is that definitive bacteriology will be obtained from the ANC/IPN by drainage (in the same way FNA was used). Further research is required to determine whether, in the absence of an early and accurate method to diagnose IPN, organ failure itself can be used as a reliable indicator for drainage, with or without proven infection.

The timing of intervention in patients with complicated AP is one of the most difficult issues for clinicians treating these patients. The widespread adoption of the step-up approach in China is noteworthy, but the optimal timing remains unclear for antibiotics, drainage, and debridement. Intravenous antibiotic treatment is commonly used in this period as a bridge between conservative and invasive treatment and can sometimes itself obviate the need of any additional intervention in selected cases [10, 30]. However, the long-term use of antibiotics may result in an increased incidence of Candida infections and antibiotic resistance [30]. The recommendation for drainage and debridement (IAP/APA) has been to wait for 4 weeks to allow demarcation because the risks are lower if the necrosis is walled off [18]. An international survey of expert pancreatologists revealed a lack of agreement on the need to postpone drainage for 4 weeks [24]. The Dutch group failed to find any relationship between the timing of catheter drainage and the outcome and concluded that waiting 4 weeks was not always necessary [16]. They have gone on to show that clinically relevant WON (largely or fully encapsulated) occurs in 43% patients within the first 3 weeks [31]. Several observational studies have also suggested that a collection does not have to be “walled off” for safe and successful catheter drainage of IPN [30, 32]. The results of this survey are in line with that trend, with only a third of respondents indicating that encapsulation was a major factor in whether to drain or not. For the timing of debridement, less than 10% of the respondents would choose an aggressive approach like immediate debridement (before or after 4 weeks) when intervention was considered necessary.

There are some important limitations to this study. While there are responses from almost all (30/34) of the provincial regions in China and from the 4 relevant medical specialties, it is not possible to prove that the results of the survey are representative of China as a whole. Those receiving the survey request were those that expressed an interest in acute pancreatitis, but not necessarily those who made clinical or policy decisions regarding its management. In the absence of any previous national data on the management of AP, it is also not possible to say to what extent the responses reflect a change in the approach to the diagnosis and treatment of local complications of ANP. The survey has highlighted the need to adopt a standardized approach to the definition of complications and interventions in this field. The “VRP” approach to describing interventions for the local complications as we used in the questionnaire, where the visualization method, route, and purpose of intervention are tightly defined, should be considered for future studies [7, 33].

## 5. Conclusion

In conclusion, there is evidence that the “step-up” approach to the management of IPN has been widely adopted in most parts of China, with PCD or endoscopic catheter drainage as the initial treatment. There is also evidence of significant variation in practice, and this serves to highlight areas warranting further research. These include the optimal way to diagnose infection; the optimal timings for antibiotics, drainage, and debridement; and the indications for intervention when infection is not suspected in relation to ANC. Whether organ failure, as a systemic complication, should be considered as an indication for local intervention in this setting warrants further investigation.

## Figures and Tables

**Table 1:** Survey questions and answers of respondents.

Survey questions	Survey answers	*N* = 321	%
(1) Would you consider drainage for sterile ANC (no elevated body temperature, white blood cell count, or PCT) without definite indications of surgical intervention (uncontrolled bleeding, perforation, suspected necrosis, aggravating compartment syndrome, etc.)?	Never	57	18
	Based on clinical condition, CT imaging, and laboratory indicators	229	71
	Immediate intervention whenever possible	35	11

(1.1) For those who consider drainage for noninfected ANC based on clinical condition, CT imaging, and laboratory indicators (*N* = 229), what is the most important?	Persistent organ failure	126	55
	Persistent abdominal pain and abdominal distention	39	17
	CT shows a larger range of ANC	34	15
	Laboratory indicators such as WBC, IL-6, CRP, and other inflammatory markers increase	18	8
	Others	12	5

(1.1.1) For those who consider drainage for noninfected ANC based on persistent organ failure(*N* = 126), what is the criteria of duration and degree of organ failure when you consider drainage? (Multiple-choice questions)	Drainage when the duration of organ failure lasts 48 h	57	45
	Drainage when the duration of organ failure lasts 1 week	21	17
	Drainage when with new-onset organ failure	41	33
	Drainage when organ failure aggravated	53	42
	Drainage when the duration of organ failure lasts 2 weeks	9	7
	Others	0	0

(2) For the drainage of ANC without infectious manifestations such as elevated body temperature, white blood cell count, or PCT, what is your first choice?	PCD	153	47
	Endoscopic catheter drainage	12	4
	Percutaneous or endoscopic catheter drainage based on the location of ANC	135	42
	Surgical drainage	12	4
	Others	9	3

(3) What is most important for the early suspicion of infected ANC?	Spiking of body temperatures to greater than 38.5°C	70	22
	New-onset organ failure or deteriorated organ failure	82	26
	Based on the imaging changes of CT or MRI	84	26
	Based on laboratory indicators, such as PCT, hemogram, and platelet	27	8
	Based on the result of fine needle aspiration	51	16
	Others	7	2

(4) Is fine needle aspiration a routine practice for diagnosing IPN in your hospital?	Yes. Nearly every suspected IPN patients will do this	25	8
	No. Only a part of patients do this	166	52
	Never	130	40

(5) If IPN is suspected, what is the most important factor determining whether you intervene or not?	Based on the result of blood culture	7	2
	Based on the occurrence and evolution of organ failure	45	14
	Based on the efficacy of antibiotic therapy	79	25
	Based on whether the disease extends beyond four weeks and the collection becomes walled off	112	35
	Immediate drainage without considering other factors	72	22
	Others	6	2

(6) If IPN is suspected or diagnosed but the course of the disease is less than 4 weeks (the collection is not well demarcated), what is your choice?	Antibiotic therapy alone	5	2
	Catheter drainage when antibiotic therapy is ineffective	51	16
	Antibiotic treatment and immediate drainage, but debride (including endoscopy or laparotomy) after 4 weeks (or wall formation) (including endoscopy or laparotomy)	162	50
	Antibiotic treatment, meanwhile wait until 4 weeks (or wall formation) and then drainage or debridement	79	25
	Immediately debridement (including endoscopy or laparotomy)	16	5
	Others	8	2

(7) If IPN is suspected or diagnosed and the course of the disease has reached 4 weeks (or with WON), what is your first choice?	Antibiotic therapy alone	2	0
	Catheter drainage when antibiotic therapy is ineffective	25	8
	Antibiotic therapy meanwhile catheter drainage. The decision of debridement is made based on the step-up approach	250	78
	Antibiotic therapy meanwhile immediate minimally invasive debridement	25	8
	Antibiotic therapy meanwhile laparotomy debridement and drainage	12	4
	Others	7	2

(8) What is the preferred drainage approach in treating IPN when intervention is considered necessary in your hospital?	Step-up approach with PCD as the initial treatment	162	50
	Step-up approach with endoscopic catheter drainage as the initial treatment	14	4
	Step-up approach with PCD or endoscopic catheter drainage as the initial treatment based on the location of ANC	118	37
	Direct laparotomy debridement and drainage	15	5
	Others	12	4

(9) For walled-off necrosis (WON) with symptoms such as persistent abdominal pain, stomach outlet obstruction, and emaciation, what is your preferred treatment?	Observation without drainage	6	2
	Step-up approach with PCD as the initial treatment	97	30
	Step-up approach with endoscopic catheter drainage as the initial treatment	24	7
	Step-up approach with PCD or endoscopic catheter drainage as the initial treatment based on the location of WON	141	44
	Direct laparotomy debridement and drainage	45	14
	Others	8	3

## Data Availability

All relevant data analyzed during this study are presented in tabular form in this published article. The original datasets used during the current study are available from the corresponding author on reasonable request.

## Supplementary Materials

Supplementary Materials Tables 1–3: answers of respondents from different departments to each question are listed.
