# Supplementary material for: The Diagnosis and Treatment of Local Complications of Acute Necrotizing Pancreatitis in China: A National Survey
Source: Gastroenterol Res Pract. 2021 Jul 10;2021:6611149. doi: 10.1155/2021/6611149 (PMC8286200; doi:10.1155/2021/6611149)
Supplement: Supplementary Materials — Tables 1–3: answers of respondents from different departments to each question are listed. [file 6611149.f1.doc]

| Table 1. Answers of respondents from different departments to Question 1-9 | | | | | | | |
| --- | --- | --- | --- | --- | --- | --- | --- |
| Question | Answer | ICU  N=197 | Gastroenterology  N=59 | General Surgery  N=44 | Emergency Medicine  N=17 | Others  N=4 | Total  N=321 |
| Q1 | A | 30 (17.8%) | 15 (25.4%) | 10 (22.7%) | 2 (11.8%) | 0 | 57 |
|  | B | 138 (71.3%) | 42 (71.2%) | 31 (70.5%) | 14 (82.4%) | 4 (100%) | 229 |
|  | C | 29 (14.7%) | 2 (3.4%) | 3 (6.8%) | 1 (5.9%) | 0 | 35 |
| Q2 | A | 101 (51.0%) | 17 (29.3%) | 22 (50.0%) | 12 (70.6%) | 1 (25.0%) | 153 |
|  | B | 7 (3.5%) | 5 (8.6%) | 0 | 0 | 0 | 12 |
|  | C | 82 (41.4%) | 33 (56.9%) | 13 (29.5%) | 5 (29.4%) | 2 (50.0%) | 135 |
|  | D | 4 (2.0%) | 3 (5.2%) | 4 (9.1%) | 0 | 1 (25.0%) | 12 |
|  | E | 4 (2.0%) | 0 | 5 (11.4%) | 0 | 0 | 9 |
| Q3 | A | 37 (18.7%) | 14 (24.1%) | 18 (40.9%) | 1 (5.9%) | 0 | 70 |
|  | B | 49 (24.7%) | 14 (24.1%) | 12 (27.3%) | 6 (35.3%) | 1 (25.0%) | 82 |
|  | C | 51 (25.8%) | 16 (27.6%) | 10 (22.7%) | 6 (35.3%) | 1 (25.0%) | 84 |
|  | D | 22 (11.1%) | 1 (1.7%) | 1 (2.3%) | 2 (11.8%) | 1 (25.0%) | 27 |
|  | E | 35 (17.7%) | 11 (19.0%) | 3 (6.8%) | 1 (5.9%) | 1 (25.0%) | 51 |
|  | F | 4 (2.0%) | 2 (3.4%) | 0 | 1 (5.9%) | 0 | 7 |
| Q4 | A | 19 (9.6%) | 2 (3.4%) | 4 (9.1%) | 0 | 0 | 25 |
|  | B | 103 (50.2%) | 33 (56.9%) | 19 (43.2%) | 8 (47.1%) | 3 (75.0%) | 166 |
|  | C | 76 (38.4%) | 23 (39.7%) | 21 (47.7%) | 9 (52.9%) | 1 (25.0%) | 130 |
| Q5 | A | 5 (2.5%) | 2 (3.4%) | 0 | 0 | 0 | 7 |
|  | B | 34 (17.2%) | 2 (3.4%) | 4 (9.1%) | 5 (29.4%) | 0 | 45 |
|  | C | 36 (18.2%) | 25 (43.1%) | 16 (36.4%) | 2 (11.8%) | 3 (75.0%) | 79 |
|  | D | 67 (33.8%) | 23 (39.7%) | 10 (22.7%) | 9 (52.9%) | 1 (25.0%) | 112 |
|  | E | 51 (25.8%) | 6 (10.3%) | 13 (29.5%) | 1 (5.9%) | 0 | 72 |
|  | F | 5 (2.5%) | 0 | 1 (2.3%) | 0 | 0 | 6 |
| Q6 | A | 4 (2.0%) | 1 (1.7%) | 0 | 0 | 0 | 5 |
|  | B | 26 (13.1%) | 13 (22.4%) | 8 (18.2%) | 4 (23.5%) | 0 | 51 |
|  | C | 107 (54.0%) | 25 (43.1%) | 18 (40.9%) | 9 (52.9%) | 3 (75.0%) | 162 |
|  | D | 48 (24.2%) | 15 (25.4%) | 11 (25.0%) | 4 (23.5%) | 1 (25.0%) | 79 |
|  | E | 8 (4.0%) | 6 (6.9%) | 4 (9.1%) | 0 | 0 | 16 |
|  | F | 5 (2.5%) | 0 | 3 (6.8%) | 0 | 0 | 8 |
| Q7 | A | 1 (0.5%) | 1 (1.7%) | 0 | 0 | 0 | 2 |
|  | B | 13 (6.6%) | 6 (10.3%) | 5 (11.4%) | 1 (5.9%) | 0 | 25 |
|  | C | 156 (78.8%) | 42 (71.2%) | 38 (86.4%) | 11 (64.7%) | 3 (75.0%) | 250 |
|  | D | 16 (8.1%) | 6 (10.3%) | 0 | 3 (17.6%) | 0 | 25 |
|  | E | 7 (3.5%) | 2 (3.4%) | 1 (2.3%) | 2 (11.8%) | 0 | 12 |
|  | F | 5 (2.5%) | 1 (1.7%) | 0 | 0 | 1 (25.0%) | 7 |
| Q8 | A | 108 (54.5%) | 19 (32.8%) | 22 (50.0%) | 12 (70.6%) | 0 | 162 |
|  | B | 8 (4.0%) | 6 (10.3%) | 0 | 0 | 1 (25.0%) | 14 |
|  | C | 68 (34.3%) | 30 (51.7) | 14 (31.8%) | 3 (17.6%) | 3 (75.0%) | 118 |
|  | D | 7 (3.5%) | 2 (3.4%) | 5 (11.4%) | 1 (5.9%) | 0 | 15 |
|  | E | 7 (3.5%) | 1 (1.7%) | 3 (6.8%) | 1 (5.9%) | 0 | 12 |
| Q9 | A | 3 (1.5%) | 1 (1.7%) | 2 (4.5%) | 0 | 0 | 6 |
|  | B | 69 (34.8%) | 7 (12.1%) | 14 (31.8%) | 7 (41.2%) | 0 | 97 |
|  | C | 11 (5.6%) | 12 (20.7%) | 0 | 1 (5.9%) | 0 | 24 |
|  | D | 83 (41.9%) | 34 (58.6) | 14 (31.8%) | 6 (35.3%) | 4 (100%) | 141 |
|  | E | 27 (13.6%) | 4 (6.9%) | 11 (31.8%) | 3 (17.6%) | 0 | 45 |
|  | F | 5 (2.5%) | 0 | 3 (6.8%) | 0 |  | 8 |

| Table 2. Answers of respondents from different departments to Question 1.1 | | | | | | | |
| --- | --- | --- | --- | --- | --- | --- | --- |
| Question | Answer | ICU  N=138 | Gastroenterology  N=42 | General Surgery  N=31 | Emergency Medicine N=14 | Others N=4 | Total  N=229 |
| Q1.1 | A | 76 (55.1%) | 24 (57.1%) | 16 (51.6%) | 7 (50.0%) | 3 (75.0%) | 126 |
|  | B | 21 (15.2%) | 7 (16.7%) | 8 (25.8%) | 3 (21.4%) | 0 | 39 |
|  | C | 20 (14.5%) | 6 (14.3%) | 5 (16.1%) | 2 (14.3%) | 1 (25.0%) | 34 |
|  | D | 13 (9.4%) | 3 (7.1%) | 1 (3.2%) | 1 (7.1%) | 0 | 18 |
|  | E | 8 (5.8%) | 2 (4.8%) | 1 (3.2%) | 1 (7.1%) | 0 | 12 |

| Table 3. Answers of respondents from different departments to Question 1.1.1 | | | | | | | |
| --- | --- | --- | --- | --- | --- | --- | --- |
| Question | Answer | ICU  N=76 | Gastroenterology  N=24 | General Surgery  N=16 | Emergency medicine  N=7 | Others N=3 | Total  N=126 |
| Q1.1.1 | A | 35 (46.1%) | 11 (45.8%) | 6 (37.5%) | 2 (28.6%) | 3 (100%) | 57 |
|  | B | 12 (15.8%) | 5 (20.8%) | 3 (18.8%) | 1 (14.3%) | 0 | 21 |
|  | C | 28 (36.8%) | 5 (20.8%) | 3 (18.8%) | 2 (28.6%) | 3 (100%) | 41 |
|  | D | 36 (47.4%) | 6 (25.0%) | 7 (43.8%) | 2 (28.6%) | 2 (66.7%) | 53 |
|  | E | 7 (9.2%) | 0 | 0 | 2 (28.6%) | 0 | 9 |
|  | F | 0 | 0 | 0 | 0 | 0 | 0 |
